# Malignant Transformation of Reese's Melanosis: A Case of Conjunctival Melanoma and Related Therapeutic Modalities

**DOI:** 10.7759/cureus.62331

**Published:** 2024-06-13

**Authors:** Ikram Kharmach, Fatima Rezzoug, Mohamed Moukhlissi, Ouissam Al Jarroudi, Sami Aziz Brahmi, Said Afqir

**Affiliations:** 1 Medical Oncology, Mohammed VI University Hospital, Oujda, MAR; 2 Medical Oncology, Faculty of Medicine and Pharmacy, Mohammed First University, Oujda, MAR; 3 Radiotherapy, Mohammed VI University Hospital, Oujda, MAR

**Keywords:** brachytherapy, mitomycin eye drops, surgical intervention, reese's melanosis, conjunctival melanoma

## Abstract

Conjunctival melanoma is a rare but aggressive condition that can arise from healthy conjunctiva, pre-existing nevi, or precancerous conditions like Reese's melanosis. This acquired primary conjunctival melanosis can significantly impact an individual's quality of life due to its potential for recurrence and metastasis. Effective treatment typically requires a multidisciplinary approach to optimize outcomes. We present the case of a 56-year-old patient with recurrent Reese melanoma who underwent multiple surgeries. During the last intervention, a malignant transformation into melanoma was discovered. Due to the absence of brachytherapy facilities, the patient received local treatment with mitomycin C eye drops. Despite this limitation, the patient showed no signs of recurrence one year post-treatment. Given the high risk of local recurrence after surgery alone, additional radiotherapy is recommended and should be systematically discussed. Regular monitoring and timely intervention are essential to prevent disease progression. Notably, the frequent BRAF (B-Raf proto-oncogene, serine/threonine kinase) mutation in conjunctival melanoma opens possibilities for targeted therapies, such as BRAF inhibitors, offering promising options for management alongside traditional surgical approaches.

## Introduction

Malignant conjunctival melanoma is a rare but potentially deadly tumor of the ocular surface [1]. It represents only 2% of all eye tumors [2], thus occupying a minority but notable proportion of non-cutaneous melanomas, estimated at around 1.6% [3]. This malignancy typically occurs around the age of 60 and rarely in young adults under 40 [1]. It has a tendency to develop on healthy conjunctiva or a pre-existing nevus, although it can also arise from a precancerous condition such as Reese's melanosis [4]. Reese's melanosis primarily affects Caucasians aged 60 and above [5]. It can involve different parts of the eye, such as the bulbar conjunctiva, fornix, tarsal conjunctiva, or even the skin of the eyelids or cornea [6]. The potential for degeneration into invasive melanoma varies depending on the case. However, retrospective studies indicate that the extent of conjunctival involvement can be used as a predictive factor for malignant transformation: lesions that cover less than 1 clock hour have a low risk, while those that involve 3 or more clock hours have a risk of over 20% for malignant transformation [7]. Clinically, it is characterized by its unilateral nature and may manifest in pigmented areas ranging from golden yellow to dark brown, appearing as spots on the conjunctiva [5]. The transformation of conjunctival melanocytic lesions into melanoma is significantly influenced by genetic alterations in genes such as BRAF, NRAS (neuroblastoma RAS viral oncogene homolog), and NF1 (neurofibromin 1). In addition, chronic sun exposure and immunosuppression are recognized as contributing factors [8]. Conjunctival melanoma has an unfavorable prognosis due to its recurrent nature and tendency to metastasize [9]. The therapeutic management of conjunctival melanoma poses challenges, both in terms of local and distant recurrence, notably at the locoregional or visceral lymph node level [10]. This treatment mainly relies on surgery, radiotherapy, and chemotherapy [5]. In this paper, we present a case of a patient who was monitored for Reese's melanosis over a period of 10 years. The patient experienced multiple recurrent episodes, which were managed through excision surgeries, until the development of malignant conjunctival melanoma.

## Case presentation

**Figure 1:**
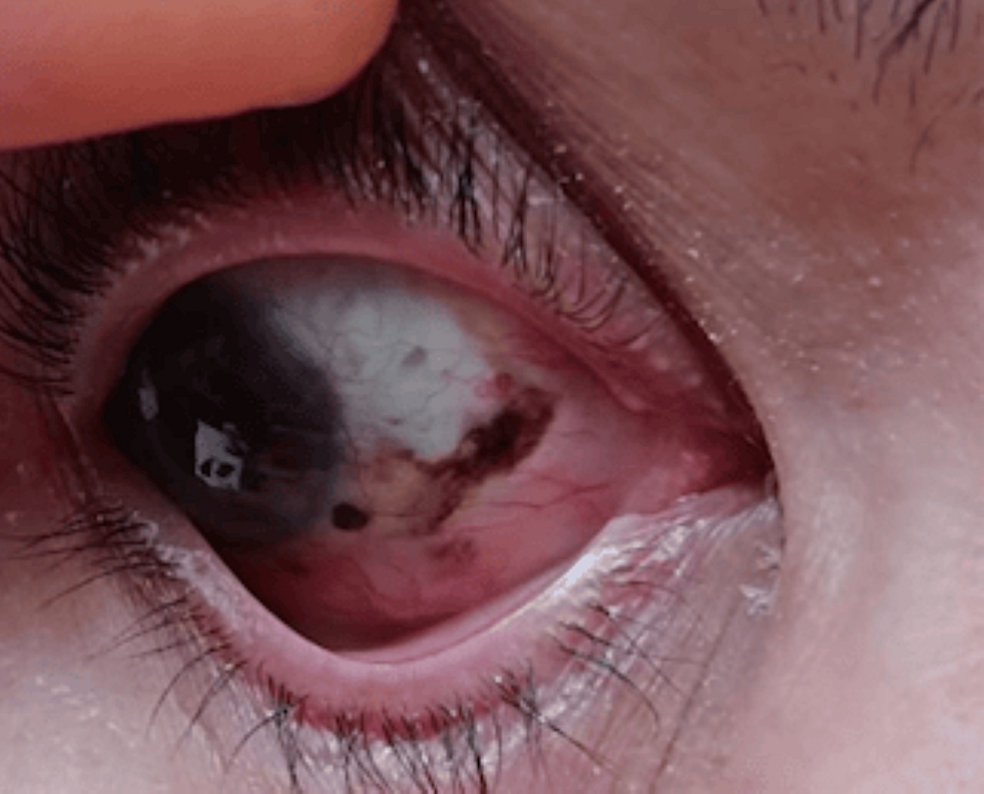
Clinical appearance of conjunctival melanosis in the patient's right eye. Pigmented lesions with irregular borders, raised and affecting the bulbar conjunctiva. Feeder vessels are also visible.

A 56-year-old Moroccan housewife, with no notable medical history, has been monitored for 10 years for Reese's melanosis. The right eye displays heterogeneous pigmentation, especially on the bulbar conjunctiva, and a small, well-defined, rounded nodule around the limbus. This anomaly has gradually increased in size and has been managed solely with excision surgeries (Figure 1). Due to our center's specialization in oncological conditions, comprehensive ocular examination findings were not included in this study. We adapt assessments to prioritize tumor evaluation and treatment response, although we acknowledge the significance of ocular evaluations in specific contexts. 

**Figure 2:**
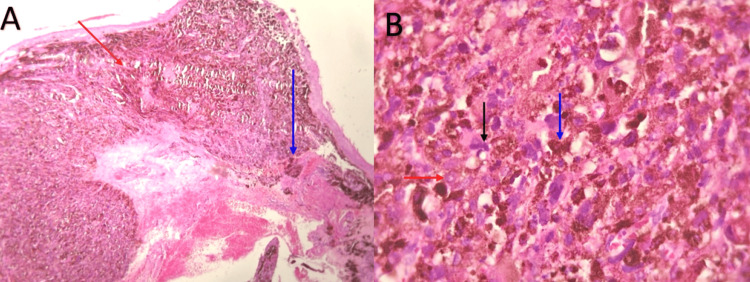
Histopathological findings of conjunctival biopsy. (A) Microscopic features showing a diffuse pigmented proliferation (red arrow), of diffuse architecture, and infiltrating the conjunctival mucosa (blue arrow) (hematoxylin and eosin, x 40). (B) Tumor cells are epithelioid with elongated polygonal cells showing hyperchromatic nuclei with prominent nucleoli (red arrow) and mitosis (black arrow). The cytoplasm has melanic pigments (blue arrow) (hematoxylin and eosin, x400).

Multiple resections of benign lesions were performed, ensuring clear surgical margins. The last intervention revealed a malignant transformation of the tumor. A histopathological examination confirmed the presence of epithelioid conjunctival malignant melanoma (Figure 2A). This type of melanoma is marked by high-density epithelioid melanocytic proliferation, with elongated polygonal cells showing hyperchromatic nuclei with prominent nucleoli and hemorrhagic suffusions. The mitotic index, assessed at 2 mitoses/10 fields (Figure 2B), did not show vascular emboli.

Following a multidisciplinary meeting, brachytherapy was proposed as a complementary treatment, but its unavailability at the hospital and the patient's limited resources prevented its implementation. Consequently, local treatment with mitomycin C eye drops was initiated. The positron emission tomography scan was suggested, but the patient couldn’t undergo it due to lack of means. A computerized tomography scan was performed and found to be normal. The patient was regularly followed up, with monitoring every three months by our oncology team for physical examinations to check for signs of disease progression or new symptoms. Additionally, her ophthalmologist conducted regular ocular examinations to monitor for any signs of recurrence. One year after these events, the patient remains well, with no recurrence or relapse, and has reported satisfaction with the treatment received.

## Discussion

Malignant conjunctival melanoma is an exceptionally rare condition, accounting for only 2% of all eye tumors [9]. It arises from primary conjunctival melanosis in 50 to 70% of cases, although it can also manifest on healthy conjunctiva, termed de novo melanoma, or, more rarely, originate from the transformation of a pre-existing conjunctival nevus [11,12]. Reese's melanosis is an acquired primary conjunctival melanosis often transformed after a variable period of evolution into a malignant melanoma, from which new tumor locations can occur remotely even if the initial tumor is cured. This is due to the involvement of the entire conjunctiva by precancerous melanosis [2]. From a histological perspective, this condition is characterized by the proliferation of atypical melanocytes along the basal layer of the epithelium, which releases pigment into the deeper dermis. Malignant transformation is characterized by vertical proliferation with a collapse at the base, but it typically occurs after a long period of evolution [6]. Reaching vascularization at the chorion level, particularly lymphatic vascularization, explains the risk of metastasis and death, estimated to be between 16% and 32% over a five-year period [3]. The treatment of acquired precancerous melanomas poses complex challenges due to the predisposition of the entire conjunctiva to develop malignant melanomas. Paridaens et al. recommended exenteration to remove all potentially affected conjunctiva [13]. However, studies have found that this approach does not always prevent the risk of distant metastasis. Therefore, exenteration is recommended only when treatment fails or a massive tumor is present [14]. The majority of authors favor treatment with cryotherapy for flat pigmented areas, combined with surgical excision of nodular lesions. Indeed, this approach reduces the risk of malignant degeneration by eliminating atypical melanocytes in conjunctival biopsies that have undergone this therapeutic strategy [15]. Therefore, it is advisable to pursue aggressive treatment for melanosis degeneration. Additional treatment is important to prevent the risk of local recurrence, even if the surgical margins are intact, as safety margins may not be as sufficient as those respected at the cutaneous level. Several authors have successfully attempted conservative treatments through a combination of surgery and radiotherapy. Haye et al. have demonstrated the effectiveness of this combination, which reduces mortality by half at 10 years compared to surgery or radiotherapy alone [16]. Radiotherapy often administers high doses to treat melanoma via brachytherapy, aiming to target the tumor while preserving the globe and vision whenever possible. Adjuvant cryotherapy targets superficial or residual melanocytes during the excision of conjunctival melanoma, significantly reducing rates of local relapse when combined with surgical excision, making it an effective therapeutic option [17]. Recent studies suggest that local chemotherapy treatments with mitomycin C eye drops can reduce or even completely eliminate conjunctival pigmentation. They appear to have some efficacy, at least temporarily [18]. When evaluating the prognosis of conjunctival carcinomas, various factors are considered, including the tumor location. Tumors located at the limbus or bulbar conjunctiva tend to have a better prognosis than those localized at the fornices, caruncles, or eyelids [2]. Other factors, such as tumor thickness exceeding 2 mm, the number of previous surgeries, and local recurrences, are also considered [19]. De novo tumors have a poorer prognosis for overall survival compared to those developed in Reese's melanosis [2]. The rates of local recurrence and mortality in the literature vary depending on the studies and the type of treatment received. At five years, local recurrence rates ranged from 26 to 50%, and mortality rates ranged from 7% to 32% [19]. Recent research has identified the BRAF V600E mutation in approximately half of primary conjunctival melanoma cases and over half of metastatic lesions. Because of these results, the BRAF inhibitor PLX4023 (vemurafenib) has been tested to see how well it treats the V600E mutation in conjunctival melanoma, using examples of skin melanomas with the same mutation [8].

## Conclusions

Eye cancers are rare but potentially serious, requiring multidisciplinary treatment to maximize the chances of a cure. Rapid and appropriate care determines the ocular and vital functional prognosis. Recent data reveal various therapeutic modalities, ranging from surgical excision to the use of targeted therapies such as BRAF inhibitors. Given the high potential for recurrence of Reese's melanosis, regular surveillance is essential for detecting recurrences.
